# Timing of exercise for muscle strength and physical function in men initiating ADT for prostate cancer

**DOI:** 10.1038/s41391-019-0200-z

**Published:** 2020-02-04

**Authors:** Robert U. Newton, Daniel A. Galvão, Nigel Spry, David Joseph, Suzanne K. Chambers, Robert A. Gardiner, Dickon Hayne, Dennis R. Taaffe

**Affiliations:** 1 Exercise Medicine Research Institute, Edith Cowan University, Joondalup, WA Australia; 2 School of Medical and Health Sciences, Edith Cowan University, Joondalup, WA Australia; 3 School of Human Movement and Nutrition Sciences, University of Queensland, Brisbane, QLD Australia; 4 Genesis CancerCare, Joondalup, WA Australia; 5 Faculty of Medicine, University of Western Australia, Nedlands, WA Australia; 6 Department of Radiation Oncology, Sir Charles Gairdner Hospital, Nedlands, WA Australia; 7 Faculty of Health, University Technology Sydney, Sydney, Australia; 8 Department of Urology, Royal Brisbane and Women’s Hospital, Brisbane, QLD Australia; 9 Centre for Clinical Research, University of Queensland, Brisbane, QLD Australia; 10 UWA Medical School, University of Western Australia, Crawley, WA Australia; 11 Fiona Stanley Hospital, Murdoch, WA Australia

**Keywords:** Cancer therapy, Outcomes research

## Abstract

**Background:**

Androgen deprivation therapy (ADT) in men with prostate cancer (PCa) results in adverse effects, including reduced muscle strength and physical function, potentially compromising daily functioning. We examined whether it was more efficacious to commence exercise at the onset of ADT rather than later in treatment to counter declines in strength and physical function.

**Methods:**

One-hundred-and-four men with PCa (68.3 ± 7.0 years) initiating ADT were randomised to immediate exercise (IMX, *n* = 54) or delayed exercise (DEL, *n* = 50) for 12 months. IMX comprised 6 months of supervised resistance/aerobic/impact exercise initiated at the onset of ADT with a 6-month follow-up. DEL comprised 6 months of usual care followed by 6 months of resistance/aerobic/impact exercise. Upper and lower body muscle strength and physical function were assessed at baseline, 6 and 12 months.

**Results:**

There was a significant difference for all strength measures at 6 months favouring IMX (*P* < 0.001), with net differences in leg press, seated row and chest press strength of 19.9 kg (95% CI, 12.3–27.5 kg), 5.6 kg (3.8–7.4 kg) and 4.3 kg (2.7–5.8 kg), respectively. From 7 to 12 months, DEL increased in all strength measures (*P* < 0.001), with no differences between groups at 12 months. Similarly, physical function improved (*P* < 0.001) in IMX compared with DEL at 6 months for the 6-m fast walk (−0.2, 95% CI −0.3 to −0.1 s), 400-m walk (−9.7, −14.8 to −4.6 s), stair climb (−0.4, −0.6 to −0.2 s) and chair rise (−1.0, −1.4 to −0.7 s), with no differences between groups by 12 months, except for the 6-m fast walk (*P* < 0.001).

**Conclusion:**

Exercise either at the onset or after 6 months of ADT preserves/enhances muscle strength and physical function. However, to avoid initial treatment-related adverse effects on strength and function, exercise therapy should be implemented with initiation of ADT.

## Introduction

Up to half of prostate cancer (PCa) patients at some stage following diagnosis will undergo androgen deprivation therapy (ADT) [1, 2]. Although beneficial as a neoadjuvant and adjuvant treatment delaying disease progression in patients with localised and advanced disease, reducing testosterone to castrate levels negatively impacts the patient’s well-being, co-morbidity risk and quality of life [3, 4]. These treatment toxicities are well established, impacting multiple body systems [5], and may be especially detrimental in older patients with compromised physical capacity and numerous comorbidities.

One adverse effect resulting from severe hypogonadism is a loss of muscle mass [6, 7], which is accompanied by a decline in muscle strength and physical function [8–12]. However, we and others have demonstrated that deficits in muscle strength [13–15] and physical function [14, 15] as a result of ADT in men with PCa can be reversed following a period of resistance training or combined resistance and aerobic training. In these trials, men were on established ADT regimens with exercise initiated with rehabilitative intent to counter treatment-related toxicities. Nevertheless, a more opportune time to intervene may be when ADT is initiated in order to prevent these treatment-related adverse effects from occurring in the first place. As a result, we previously undertook a short-term 3-month exercise trial in men commencing hormone suppression, and were able to prevent declines in muscle strength and function [16]. In the trial [16], men were randomised to supervised twice-weekly moderate-to-high-intensity aerobic and resistance training, which was undertaken in an exercise clinic or to usual care. There were significant net differences between groups at 3 months for upper and lower body muscle strength, as well as for repeated chair rise test and 400-m walk, with improved strength and performance in the exercise, and poorer strength and performance in the usual care group.

In this report, we extend the findings from our previous work [14–16], and address whether it is more efficacious to prevent ADT-related declines in strength and function from the outset rather than undertaking exercise to rehabilitate the patient following their development. In this 12-month randomised trial, men either concurrently initiated exercise and ADT for 6 months (with no supervised exercise in the following 6 months) or delayed undertaking exercise until after 6 months of ADT treatment. We hypothesised that exercise concurrently initiated with ADT would prevent deficits in muscle strength and physical function from occurring; however, by 12 months there would be no difference compared with the delay group where exercise was commenced with rehabilitative intent. No difference between groups at 12 months was based on anticipated improvements in the delay group, with partial reversal of gains exhibited in the group that undertook immediate exercise (IMX) [17].

## Materials and methods

### Patients

Two-hundred-and-nineteen PCa patients were referred by their treating radiation oncologist/urologist and screened for participation from August 2013 to April 2015 in Perth, Western Australia, as previously reported [18]. Inclusion criteria included commencing ADT and intending to remain on it for at least the next 6 months, no structured aerobic or resistance exercise in the past 3 months and able to walk 400 m. Exclusion criteria included prior ADT, established metastatic disease, established osteoporosis, medications known to affect bone metabolism, acute illness or any musculoskeletal, cardiovascular or neurological disorder that could inhibit or put them at risk from exercising as determined by their physician. Following screening, 115 patients were excluded mainly due to already-commenced ADT (*n* = 29), too far to travel (*n* = 19), declined to participate (*n* = 17), health issues (*n* = 13), no time for training (*n* = 12), going on holidays (*n* = 8) and unwilling to be randomised (*n* = 6). All the remaining participants obtained medical clearance from their physician. The study was approved by the Edith Cowan University Human Research Ethics Committee, and all participants provided written informed consent.

### Study design

One-hundred-and-four men were randomly assigned by computer-generated number sequence to IMX or delayed exercise (DEL), stratified according to age (≤70 and >70 years) and smoking status (yes/no) [18, 19]. Investigators and research assistants performing the assessments were blinded to group allocation. The primary endpoint was bone mineral density, which we have recently reported [18], with secondary endpoints including muscle strength and physical function. IMX undertook a programme that combined resistance + impact loading + aerobic exercise for the initial 6 months, with no formal intervention in the second 6-month period. DEL underwent 6 months of usual care followed by the identical exercise programme in the second 6-month period. Participants received daily supplementation with calcium (1000 mg/d) and vitamin D_3_ (800 IU/d) throughout the study period. Measurements were performed at baseline, 6 and 12 months.

### Exercise programme

The exercise programme has been previously described in detail [18, 19]. In brief, the multicomponent exercise programme was undertaken thrice weekly in several exercise clinics, and designed to primarily target the musculoskeletal system consisting of resistance exercise and impact-loading activities, as well as aerobic-based activity for cardio-metabolic health. It was structured such that aerobic and resistance exercise was alternated weekly so that two resistance/impact loading and one aerobic/impact-loading session was performed for 1 week with the reverse performed the following week. Resistance training consisted of upper and lower body exercises that targeted the major muscle groups, and included leg press, leg extension, leg curl, chest press, seated row, lat pulldown and biceps curl, at an intensity of 6–12 RM (the maximal weight lifted 6–12 times) for 2–4 sets per exercise. Impact loading consisted of a series of bounding, hopping, skipping, leaping and drop-jumping activities, with the volume and intensity progressively increased over the 6-month training period. The aerobic-based component consisted of walking/jogging on a treadmill, and cycling or rowing on a stationary ergometer at an intensity of 60–85% estimated maximum heart rate for 25–40 min, with heart rate monitored by using chest straps and heart rate watches (Polar Electra Oy, Finland). Sessions were ~60 min in duration, undertaken in small groups and supervised by an exercise physiologist. Each session commenced with a warm-up comprising aerobic activities and concluded with stretching activities. To further load the skeleton, participants were encouraged to undertake twice-weekly home-based training consisting of a modified version of the impact-loading programme, and aerobic activities such as walking or cycling.

### Muscle strength and physical function

Dynamic concentric muscle strength for the chest press, leg press and seated row exercises was assessed using the one-repetition maximum method [20]. Physical function was determined by a battery of performance tests [14, 15, 21], and included the usual and fast 6-m walk (as measures of gait speed), 6-m backwards tandem walk (dynamic balance), 400-m walk (cardiorespiratory fitness and walking endurance), stair climb (lower body power) and repeated chair rise test (lower body function). Tests were performed in triplicate, except for the 400-m walk that was performed once, with the fastest time used in the analyses. Participants completed a familiarisation session to become accustomed to each of the test protocols prior to the first testing session.

### Other measures

Height and weight were assessed using a stadiometer and electronic scales, respectively, with body mass index (BMI, kg/m^2^) calculated. Body fat percentage was determined by dual-energy X-ray absorptiometry (Hologic Discovery A, Waltham, MA, USA). Physical activity was assessed by the Leisure Score Index of the Godin Leisure-Time Exercise Questionnaire [22]. Testosterone and prostate-specific antigen (PSA) were measured commercially by an accredited Australian National Association of Testing Authorities laboratory (Pathwest Diagnostics, Perth, WA).

### Statistical analyses

Data were analysed using IBM SPSS Version 24 (IBM Corp., Armonk, NY, USA). Normality of the distribution was assessed using the Kolmogorov–Smirnov test. Between-group differences in baseline characteristics were assessed using independent *t* tests or the Mann–Whitney U test, as appropriate, for continuous data, and *χ*^2^ for categorical data. Changes in muscle strength and physical function were assessed using analysis of covariance (baseline value as a covariate), with change from 0 to 6 months, 7 to 12 months and 0 to 12 months assessed. Data that were not normally distributed were log transformed (ln) for analysis. Intention-to-treat was utilised for analyses using maximum likelihood imputation of missing values (expectation maximisation). Baseline to 12-month comparisons for strength and function within groups were assessed using either paired *t* tests or the Wilcoxon signed-rank test. Freidman’s ANOVA with Bonferroni-adjusted Wilcoxon signed-rank test as the follow-up test was used for other measures with three time points. Tests were two-tailed, and to adjust for multiple comparisons, statistical significance was set at an *α* level of 0.01. Values are reported as the mean ± SD or median and interquartile range (IQR).

## Results

There was no difference in any demographic or clinical characteristics between groups at baseline (Table 1). Men were aged 48–84 years and 6.0 ± 2.0 days since the first treatment injection (Lucrin or Zoladex). Six men in IMX discontinued the intervention and 13 men in DEL withdrew during the initial 6 months with an additional participant from IMX lost to follow-up and 5 from DEL withdrawing by 12 months [18]. The main reasons for discontinuing exercise were unrelated injury (*n* = 2), health issues (*n* = 2) and no longer interested (*n* = 2), and for those lost to follow-up were health issues (*n* = 6), wanted to begin exercise (*n* = 3) and no longer interested (*n* = 2). As previously reported [18], treatment alterations occurred such that during the initial 6 months, 10 men in IMX and 5 in DEL ceased ADT, and 40 men in IMX and 30 men in DEL commenced radiotherapy that continued into the initial portion of the second 6-month period for 11 men in IMX and 8 in DEL. For months 7–12, an additional 19 men from IMX and 17 from DEL ceased ADT, while 1 participant in IMX recommenced ADT, with 4 additional men from IMX and 2 from DEL initiating radiotherapy. In addition, 8 men in IMX and 4 in DEL who previously had external beam radiation underwent brachytherapy during the second 6-month period. Patients in IMX attended 79% of the scheduled exercise sessions, and those in DEL attended 69% of scheduled sessions. In regard to the home-based programme, IMX completed 29 ± 17 impact and 31 ± 16 aerobic sessions, while DEL completed 22 ± 20 impact and 33 ± 13 aerobic sessions, with no difference between groups for either component (impact, *P* = 0.233; aerobic, *P* = 0.754). More detailed explanation of retention and adherence is provided in our previous paper [18]. There were no major adverse events related to the training programme.

**Table 1:** Participant characteristics.

	IMX (*n* = 54)	DEL (*n* = 50)	*P* value
Age, year	69.0 ± 6.3	67.5 ± 7.7	0.266
Height, cm	173.4 ± 7.1	172.7 ± 6.2	0.602
Weight, kg	82.9 ± 16.4	84.6 ± 12.9	0.551
BMI, kg/m^2^	27.5 ± 4.4	28.3 ± 3.9	0.282
Body fat (%)	29.0 ± 5.1	30.4 ± 5.2	0.159
Married, *N* (%)	42 (77.8)	41 (82.0)	0.914
Currently employed, *N* (%)	13 (24.1)	17 (34.0)	0.264
Tertiary education, *N* (%)	13 (24.1)	11 (22.0)	0.802
Current smoker, *N* (%)	5 (9.3)	3 (6.0)	0.533
Number of medications	3.0 (2.0–5.0)	3.0 (2.0–5.3)	0.715
Godin LSI, median (IQR)	10.0 (0.0–24.5)	10.0 (0.0–27.0)	0.872
Gleason score	7.6 ± 1.0	7.6 ± 0.8	0.829
PSA, ng/mL median (IQR)	3.4 (0.7–6.4)	3.9 (0.2–10.0)	0.599
Testosterone, nmol/L median (IQR)	8.0 (2.0–16.0)	4.6 (1.9–15.8)	0.413
Time since ADT injection (days)	6.4 ± 2.1	5.7 ± 1.9	0.110
Prostatectomy, *N* (%)	15 (27.7)	17 (34.0)	0.532
Radiation, *N* (%)	4 (7.4)	3 (6.0)	0.775
Number of comorbidities^a^	1.0 (1.0–2.0)	1.5 (1.0–2.0)	0.646

Values are the mean ± SD unless stated otherwise.

*IMX* immediate exercise, *DEL* delayed exercise, *BMI* body mass index, *LSI* Leisure Score Index with a moderate-to-strenuous LSI ≥ 24 classed as active and ≤23 classed as insufficiently active, *PSA* prostate-specific antigen, *ADT* androgen deprivation therapy, *IQR* interquartile range.

^a^Cardiovascular disease, hypertension, diabetes and dyslipidaemia.

### Muscle strength

There were no differences between groups at baseline for any muscle strength measure (*P* = 0.379–0.825). Following initiation of ADT and exercise, strength improved (*P* < 0.001) for the chest press, leg press and seated row exercises in IMX compared with DEL with adjusted differences of 4.3 kg, 19.9 kg, and 5.6 kg, respectively (Table 2). During this period, the strength declines were modest in DEL being 7% for the chest press and 4% for the seated row. Following exercise in DEL from 7 to 12 months, muscle strength increased in all three exercises (*P* < 0.001) with comparable gains to that observed for IMX during the initial 6 months such that by 12 months there were no differences between groups (*P* = 0.160–0.971). At 12 months, upper and lower body strength was significantly greater (*P* < 0.01) than at baseline for IMX and DEL.

**Table 2:** Muscle strength at baseline, 6 and 12 months.

	Baseline	6 months	12 months	0–6 months^d^		7–12 months^e^		0–12 months^f^	
				Adj. Diff.	*P* value^c^	Adj. Diff.	*P* value^c^	Adj. Diff.	*P* value^c^
Chest press (kg)^a^									
IMX	43.4 ± 12.8	44.7 ± 12.5	46.3 ± 12.1	4.3 (2.7, 5.8)	<**0.001**	−5.2 (−7.1, −3.3)	<**0.001**	−1.5 (−3.9, 1.0)	0.160
DEL	46.0 ± 16.3	42.8 ± 14.7	49.8 ± 13.9						
Leg press (kg)^a^									
IMX	119.0 ± 49.9	138.8 ± 51.6	142.8 ± 55.4	19.9 (12.3, 27.5)	<**0.001**	−18.7 (−25.0, −12.5)	<**0.001**	1.7 (−8.0, 11.4)	0.937
DEL	119.6 ± 48.4	119.5 ± 48.0	141.7 ± 51.4						
Seated row (kg)^b^									
IMX	73.9 ± 16.1	76.4 ± 15.2	77.3 ± 15.8	5.6 (3.8, 7.4)	<**0.001**	−5.9 (−7.7, −4.1)	<**0.001**	0.0 (−2.3, 2.4)	0.971
DEL	75.6 ± 16.6	72.5 ± 14.2	78.8 ± 16.7						

Values are the mean ± SD.

*IMX* immediate exercise, *DEL* delayed exercise, *Adj. Diff.* ANCOVA adjusted mean difference and 95% CI.

^a^IMX *n* = 53, DEL *n* = 48.

^b^IMX *n* = 53, DEL *n* = 49.

^c^*P* value based on log transformed data for chest press and leg press.

^d^6 months adjusted for baseline value.

^e^12 months adjusted for 6-month value.

^f^12 months adjusted for baseline value.

### Physical function

At baseline, there were no differences between IMX and DEL (*P* = 0.222–0.920). Following 6 months exercise in IMX, performance improved compared with DEL (*P* < 0.001) for the fast 6-m walk, 400-m walk, stair climb, and repeated chair rise test (Table 3). Detrimental changes in performance for DEL during the non-exercise period were modest at 2–7% for the walking tests and stair climb. Following exercise in DEL from 7 to 12 months, performance improved (*P* ≤ 0.001) for the 400-m walk, stair climb, and repeated chair rise with no difference between IMX and DEL at 12 months for any measure except for the 6-m fast walk where the modest adjusted difference of −0.2 s remained significant (*P* < 0.001) favouring IMX. Compared with baseline, at 12 months, repeated chair rise was significantly faster (*P* < 0.001) in both IMX and DEL, while stair climb time (*P* = 0.007) and 6-m fast-walk time (*P* = 0.005) were also improved in IMX. In contrast, usual 6-m walk time was slower (*P* < 0.01) in both IMX and DEL, as was 6-m fast-walk time (*P* < 0.001) in DEL.

**Table 3:** Physical function at baseline, 6 and 12 months.

	Baseline	6 months	12 months	0–6 months^c^		7–12 months^d^		0–12 months^e^	
				Adj. Diff.	*P* value^b^	Adj. Diff.	*P* value^b^	Adj. Diff.	*P* value^b^
6-m usual walk (s)									
IMX	4.2 ± 0.6	4.4 ± 0.6	4.3 ± 0.6	−0.03 (−0.2, 0.1)	0.409	−0.1 (−0.2, 0.1)	0.348	−0.1 (−0.2, 0.1)	0.121
DEL	4.2 ± 0.7	4.4 ± 0.5	4.5 ± 0.6						
6-m fast walk (s)									
IMX	3.2 ± 0.5	3.1 ± 0.5	3.1 ± 0.5	−0.2 (−0.3, −0.1)	<**0.001**	0.0 (−0.1, 0.1)	0.789	−0.2 (−0.3, −0.1)	<**0.001**
DEL	3.2 ± 0.6	3.3 ± 0.6	3.3 ± 0.6						
6-m backwards tandem walk (s)^a^									
IMX	13.2 ± 4.1	13.7 ± 4.7	12.9 ± 3.9	−0.6 (−2.2, 1.1)	0.527	−0.4 (−1.7, 0.9)	0.406	−0.6 (−1.9, 0.7)	0.643
DEL	13.9 ± 5.3	14.9 ± 7.2	14.1 ± 6.1						
400-m walk (s)									
IMX	246.8 ± 36.1	241.9 ± 37.8	245.8 ± 40.4	−9.7 (−14.8, −4.6)	<**0.001**	9.9 (5.0, 14.8)	<**0.001**	0.5 (−5.5, 6.4)	0.981
DEL	258.7 ± 53.8	263.7 ± 56.9	257.7 ± 57.5						
Stair climb (s)									
IMX	4.8 ± 1.3	4.6 ± 1.3	4.7 ± 1.3	−0.4 (−0.6, −0.2)	<**0.001**	0.4 (0.2, 0.6)	<**0.001**	0.0 (−0.1, 0.2)	0.712
DEL	5.0 ± 1.5	5.2 ± 1.6	4.8 ± 1.4						
Repeated chair rise (s)^a^									
IMX	10.7 ± 2.2	9.9 ± 1.8	9.8 ± 1.7	−1.0 (−1.4, −0.7)	<**0.001**	0.6 (0.2, 0.9)	**0.001**	−0.2 (−0.6, 0.1)	0.116
DEL	11.4 ± 2.9	11.4 ± 2.5	10.1 ± 2.1						

Values are the mean ± SD.

*IMX* immediate exercise, *DEL* delayed exercise, *Adj. Diff.* ANCOVA adjusted mean difference and 95% CI.

^a^IMX *n* = 54, DEL *n* = 49.

^b^*P* value based on log transformed data except for 6-m usual walk at 7–12 months.

^c^6 months adjusted for baseline value.

^d^12 months adjusted for 6-month value.

^e^12 months adjusted for baseline value.

### Other measures

PSA levels were reduced (*P* < 0.001) following administration of ADT in IMX and DEL at 6 [IMX, median (IQR) 0.1 (0.0–0.2); DEL 0.1 (0.0–0.4) ng/ml] and 12 months [IMX, 0.0 (0.0–0.1); DEL 0.1 (0.0–0.2) ng/ml]. Similarly, testosterone was reduced at 6 months (*P* < 0.001) [IMX, 0.5 (0.5–0.7); DEL 0.5 (0.4–0.7) nmol/L] but not 12 months due to treatment alterations [IMX, 6.4 (0.6–11.8); DEL 7.1 (1.1–13.5) nmol/L]. As expected, physical activity levels increased in IMX at 6 and 12 months (*P* = 0.001), and in DEL over the year-long trial (*P* = 0.033), although the source of the differences was not detected in follow-up testing.

## Discussion

This year-long trial comparing immediate vs. DEL in men with PCa commencing ADT produced three important findings: (1) commencing targeted exercise at the onset of ADT not only preserved but improved muscle strength and physical function in the initial treatment period; (2) losses that did occur in strength and function in DEL during the initial 6 months were recouped with further gains derived following training; (3) by 12 months, muscle strength and physical function were comparable in IMX and DEL. These results indicate that initiating exercise either at the onset of ADT or after 6 months of treatment is effective, both for not only preserving but also enhancing muscle strength and physical function. However, to avoid any treatment-related declines in muscle strength and function following the initiation of ADT, exercise should be commenced at the onset of treatment.

There were significant differences between groups in both upper and lower body strength at 6 months, such that gains were observed in IMX with modest losses in upper body strength in DEL and lower body strength remaining unchanged. Moreover, the magnitude of the adjusted difference in all three strength measures was similar to our previous 3-month trial in men initiating ADT [16]. Of interest is that in our previous trial, there was also little change in leg press strength for men initiating ADT and undergoing usual care, whereas chest press and seated row strength declines of 6 and 3% were similar to the declines in DEL of 7% and 4%, respectively. In addition, in a previous cross-sectional study we undertook in men on ADT we observed that although upper body strength was adversely affected, the results were mixed for lower body strength with leg extension but not leg press strength reduced compared with healthy controls [11].

It may well be that upper body strength is more susceptible to the adverse effects of ADT-induced hypogonadism than lower body musculature, which is more subject to higher daily usage and intensity via walking, climbing stairs or rising from a seated position, and leisure-based activities such as jogging or cycling. In any event, declines in strength that occurred in DEL during the initial 6 months were recouped with further gains occurring such that by 12 months there were no differences between groups. Moreover, upper and lower body strength at 12 months was significantly greater than that at baseline in both groups, which would provide a greater reserve capacity for the performance of daily activities, helping to prolong independent living and potentially survival [23–26].

Also of interest is that the muscle strength gains in IMX at 6 months were not only preserved at 12 months, but there were also additional modest gains, although these were not statistically significant. This may be due to activity undertaken by the men during this non-supervised period, although this was not monitored as we were interested in determining if any residual effect would have occurred from the supervised period without additional tools or strategies regarding exercise participation.

As with muscle strength, exercise not only prevented declines but also enhanced physical function in most measures in IMX relative to DEL at 6 months, with no difference following training in DEL at 12 months, except for the 6-m fast walk that continued to favour IMX. Again, adverse changes in DEL were modest with initiation of ADT and similar to our previous 3-month trial [16]. In contrast, the differences we observed in our cross-sectional report [11] for the same performance battery in men on established ADT compared with healthy age-matched men were larger ranging from 5 to 20%. The short-term duration of ADT may partially account for the modest declines in function observed in DEL. In a cross-sectional study by Clay et al. [9], physical function was lower in men on long-term ADT (≥6 months) but not short-term ADT. In a similar fashion, Levy et al. [27] reported longer chair rise time in men on chronic ADT compared with men on acute ADT and non-ADT men at baseline of a 2-year study, and that men in both ADT groups had greater declines in 4-m walking velocity over the follow-up period compared with non-ADT men.

Preserving physical function is important as it may enhance patient outcomes and survival. For instance, in the Health ABC Study of older community-dwelling adults, poorer 400-m walk performance, which is related directly to maximal oxygen consumption [28], was associated with mortality, cardiovascular disease, mobility limitation and disability [29], while Cesari et al. [30] reported that poor physical performance was predictive of lower extremity limitation, hospitalization, and death. Moreover, in a recent systematic review in cancer patients, poorer physical performance was associated with treatment-related complications and poorer survival [31]. Consequently, the undertaking of exercise, preferably that is targeted and supervised [32], should be recommended to patients commencing ADT, especially those with comorbidities and poorer strength and function who may have little reserve capacity for the performance of daily functioning.

Due to changes in treatment, testosterone was actually higher at 12 months compared with 6 months in both groups. Whether this contributed to performance improvements through anabolic facilitation of training adaptation for the DEL group can only be speculated. However, this may account in part for the total reversal of the ADT-driven performance decline of this group being equivalent to IMX at 12 months.

There are several strengths of this study. First, we compared exercise with preventative vs. rehabilitative intent, which is the customary way that exercise is administered to this patient group, to counter ADT-related adverse effects on strength and function. Second, we included both upper and lower body dynamic muscle strength, as well as several objective performance measures capturing different components of physical function. Third, patients undertook a supervised multicomponent program that was designed to counter a range of musculoskeletal and cardio-metabolic treatment-related toxicities. However, there are limitations in that a few patients in both groups also underwent radiotherapy during the trial, a result largely of being referred by radiation oncologists. In addition, several men in both groups had their treatment altered during the trial, which reflects the nature of treatment changes based on patient responses, disease progression, and developments in clinical practice. It needs to be noted that a relatively high attrition of 36% occurred in DEL, although those who dropped out were not distinguished from those who completed the trial for any baseline characteristics, including muscle strength and physical function. The patients in this study may not represent all men with PCa undergoing ADT as they have agreed to participate in an exercise intervention.

In conclusion, implementing exercise in PCa patients initiating ADT not only preserves but enhances muscle strength and physical function, despite their compromised hormonal status. Similarly, exercise commenced after the initial period of ADT and undertaken with rehabilitative intent is beneficial for the PCa patient in recouping losses and enhancing strength and function. Nevertheless, it would appear that initiating exercise therapy at the onset of treatment should be prescribed in order to not only prevent the development of any adverse effects, but also to enhance muscle strength and physical function, and potentially progression of comorbidities. Future research should be directed to comparing more targeted exercise prescription, addressing specific issues of individual patients resulting from ADT, for example, muscle loss, fat gain and bone loss, rather than the generic multimodel programme evaluated in this study.
